# Signal Processing in Functional Near-Infrared Spectroscopy (fNIRS): Methodological Differences Lead to Different Statistical Results

**DOI:** 10.3389/fnhum.2017.00641

**Published:** 2018-01-08

**Authors:** Mischa D. Pfeifer, Felix Scholkmann, Rob Labruyère

**Affiliations:** ^1^Rehabilitation Center for Children and Adolescents, University Children’s Hospital Zurich, Affoltern am Albis, Switzerland; ^2^Biomedical Optics Research Laboratory, Department of Neonatology, University Hospital Zurich, University of Zurich, Zurich, Switzerland; ^3^Children’s Research Center, University Children’s Hospital Zurich, Zurich, Switzerland

**Keywords:** optical neuroimaging, neurovascular coupling, scalp blood flow, systemic hemodynamics, signal contamination, nirsLAB, robotics, AtlasViewer

## Abstract

Even though research in the field of functional near-infrared spectroscopy (fNIRS) has been performed for more than 20 years, consensus on signal processing methods is still lacking. A significant knowledge gap exists between established researchers and those entering the field. One major issue regularly observed in publications from researchers new to the field is the failure to consider possible signal contamination by hemodynamic changes unrelated to neurovascular coupling (i.e., scalp blood flow and systemic blood flow). This might be due to the fact that these researchers use the signal processing methods provided by the manufacturers of their measurement device without an advanced understanding of the performed steps. The aim of the present study was to investigate how different signal processing approaches (including and excluding approaches that partially correct for the possible signal contamination) affect the results of a typical functional neuroimaging study performed with fNIRS. In particular, we evaluated one standard signal processing method provided by a commercial company and compared it to three customized approaches. We thereby investigated the influence of the chosen method on the statistical outcome of a clinical data set (task-evoked motor cortex activity). No short-channels were used in the present study and therefore two types of multi-channel corrections based on multiple long-channels were applied. The choice of the signal processing method had a considerable influence on the outcome of the study. While methods that ignored the contamination of the fNIRS signals by task-evoked physiological noise yielded several significant hemodynamic responses over the whole head, the statistical significance of these findings disappeared when accounting for part of the contamination using a multi-channel regression. We conclude that adopting signal processing methods that correct for physiological confounding effects might yield more realistic results in cases where multi-distance measurements are not possible. Furthermore, we recommend using manufacturers’ standard signal processing methods only in case the user has an advanced understanding of every signal processing step performed.

## Introduction

Optical neuroimaging based on functional near-infrared spectroscopy (fNIRS) is a technique increasingly used to perform neuroscientific studies. fNIRS allows to measure changes in tissue hemodynamics (blood perfusion) and oxygenation on the human head non-invasively (Scholkmann et al., 2014). Compared to other neuroimaging modalities, fNIRS offers distinct advantages: while functional magnetic resonance imaging (fMRI) has a higher spatial resolution and can reach subcortical areas, fNIRS is easier to use, in a lower price segment, and potentially portable (Cutini and Brigadoi, 2014; Piper et al., 2014; Scholkmann et al., 2014; Nieuwhof et al., 2016). Compared to electroencephalography (EEG), fNIRS can provide higher spatial resolution, is user-friendlier, and more robust to head movement (Perrey, 2008; Cutini and Brigadoi, 2014; Scholkmann et al., 2014; Nieuwhof et al., 2016). With the growing popularity of fNIRS and the increasing availability of commercial devices at decreasing costs, more and more researchers start using this technique (Boas et al., 2014) and this trend is especially observable in the fields of rehabilitation research, psychology and sport science. This observation is accompanied by an according increase in publications in the respective fields.

Currently, no standardized and widely accepted signal processing method for fNIRS exists and no fNIRS guidelines article has been published yet, in contrast to fMRI for example (Strother, 2006; Poldrack et al., 2008; Caballero-Gaudes and Reynolds, 2017). This can create the situation that novice users might mainly perform signal processing and data analysis with the tools provided by the commercial companies (like a “black box”) which could lead to “false positives” or “false negatives” in the results (Tachtsidis and Scholkmann, 2016). In both cases, one major problematic aspect are contaminations of the measured hemodynamic signal by task-evoked hemodynamic changes *not* due to neurovascular coupling in the extracerebral (scalp blood flow) as well as cerebral tissue layer (systemic blood flow) (Leff et al., 2011; Saager et al., 2011; Takahashi et al., 2011; Kirilina et al., 2012; Scholkmann et al., 2014). In the aforementioned research fields, this can be problematic for two reasons: (i) Blood flow changes in the extracerebral and cerebral layers of the head (e.g., via heart rate, blood pressure, sympathetic activation) can be already evoked by small body movements (e.g., finger tapping; Yamada et al., 2012) or psychophysiological influences. Accordingly, fNIRS studies in psychology, and especially rehabilitation research and sport science, where we often deal with persons in motion, are generally at a high risk of showing the described contamination of the hemodynamic signal. (ii) Many commercial devices currently have a fixed probe holder in their standard setup that does not enable short-distance/multi-distance measurements. These can often, if at all, only be realized with additionally purchased flexible probe holders. However, short-distance/multi-distance measurements are currently among the best methods to eliminate the signal contamination by task-evoked hemodynamics not due to neurovascular coupling (Scarpa et al., 2013; Scholkmann et al., 2014; Nambu et al., 2017a,b) and will arguably be standard in a few years’ time (Yücel et al., 2017). Short-distance/multi-distance measurements allow for separating signals that stem from blood flow changes in the extracerebral layers of the head (via short-detector separation channels, with 0.5–1.0 cm source-detector separation) from the desired neurovascular coupling-related signals of the cerebral tissue layer (via long source-detector separation channels, with 3–4 cm separation; Gagnon et al., 2011; Yamada et al., 2012; Umeyama and Yamada, 2014; Yücel et al., 2015). By using the short-detector separation channels as a reference, the components of superficial interference in the long source-detector separation channels can be regressed out (Zhang et al., 2015).

However, if no short-detector separation is possible, other approaches can and should be considered in order to remove unwanted sources from the hemodynamic response. Examples of such methods are the Principal Component Analysis (PCA; Zhang et al., 2005) and Independent Component Analysis (ICA; Kohno et al., 2007; Santosa et al., 2013), where the raw signal is decomposed into various subcomponents, assuming orthogonality (PCA)—or maximal statistical independence (ICA)—between components. Another method is the regression of the combination of long-channels from the single channels and that approach has been used in this article.

The current study explores whether different signal processing methods applied to data from a clinical fNIRS protocol without short-distance channels leads to differences in outcome. By experience, the setting without short-distance channels is still frequently used, especially in the above-mentioned research fields. We compared the standard signal processing method of nirsLAB (a freeware that comes with all instruments from NIRx Medical Technologies, Glen Head, NY, USA) to three simple customized alternative signal processing methods. Since nirsLAB has limited possibilities regarding artifact removal and filtering, nirsLAB was compared to: (i) a self-implemented signal processing method with data-adaptive filtering and advanced artifact removal; and (ii) the same self-implemented signal processing method together with an additional multi-channel regression whereby two different types of signal regression were investigated. These regressions remove a component from the signal that is common in multiple channels and that potentially reflects scalp blood flow. Our aim was to investigate whether the type of signal processing method had an impact on the final study results.

## Materials and Methods

### Subjects

A convenience sample of 15 right-handed healthy adults (4 men, 11 women), mean age ± standard deviation (SD): 29.9 ± 5.5 years (range: 24–41 years) was included in this study. Inclusion criteria were right-handedness (i.e., they had to write with the right hand) and being of age in the range 18–65 years. Exclusion criteria were having neurological disorders, or having a limiting injury of both hands or index fingers. All subjects provided written informed consent in accordance with the Declaration of Helsinki and the study and protocol was approved by the Ethics Committee of the Canton of Zurich.

### Instrumentation

#### Finger-Hand Rehabilitation Device (Amadeo)

The protocol consisted of performing passive, assisted and active index finger movements at a low frequency inside the Amadeo (Tyromotion GmbH, Graz, Austria), a finger-hand rehabilitation device with different training options for varying degrees of finger/hand impairment. Its main console consists of guide trails for each finger, which are aligned with the direction of motion of each finger. The arm of the subject is mounted into the robot by fixating the arm with cuffs and attaching the user’s fingers to these guide trails with magnets taped to the fingers. This allows each finger to be moved individually. Movements can be passive (i.e., the device performs the movement for the subject), assisted (i.e., the device assists the subject in performing the movement) and active (i.e., the subject performs the movement).

#### Functional Near-Infrared Spectroscopy

**Figure 1:**
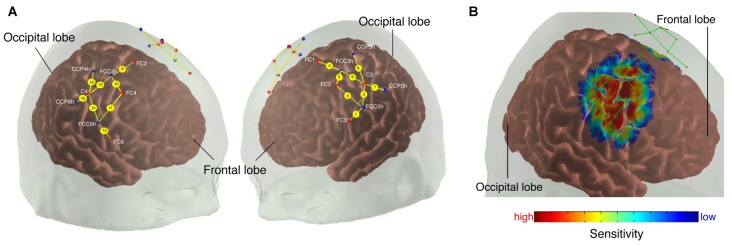
**(A)** Probe array. The colored dots indicate the light sources (red), detectors (blue) and the yellow lines represent the channels. White text indicates 10/20 electroencephalography (EEG) positions. **(B)** Sensitivity profile. The probe array displays the channels (green lines) and the optodes (red dots). The sensitivity values are displayed in log10 units.

To measure relative changes in oxygenation levels in the sensorimotor and premotor cortex (PMC) during the tasks, we used a continuous-wave NIRS system (NIRSport 8x8, NIRx Medical Technologies LLC, Berlin, Germany). NIRSport employs eight light-sources and eight detectors which can be placed into a textile EEG cap (EASYCAP, Herrsching, Germany) using the International 10/20 system for EEG recording (Chatrian et al., 1985). Each light source contains two LEDs that emit at 760 nm and 850 nm. Further information about the device can be found elsewhere (Piper et al., 2014; Vrana et al., 2016). Textile EEG caps of different sizes (i.e., circumferences of 54, 56, 58 and 60 cm) were used to take the participants’ head anatomy into account (for head circumferences between two cap sizes, the smaller cap was used). The placement of the optodes within the caps was done in reference to other studies, which used multi-channel fNIRS to assess topographic cortical activity maps (Miyai et al., 2001; Harada et al., 2009). The optodes were placed around FC3/FC4 (likely to overlie the PMC, Brodmann area 6, see Figure 1) and C3/C4 (likely to overlie the pre- and postcentral gyrus covering the primary sensorimotor cortex (M1/S1; Blatow et al., 2011, Brodmann area 4). Sources and detectors were distributed bilaterally to define 16 channels around those regions of interest (ROI), each adjacent pair of sources and detectors defining one channel (source detector separation: 25–45 mm). Eight channels were defined for each hemisphere, four channels for each PMC and M1. The focus of this study was on M1/S1, however, channels around FC3/FC4 were used for the multi-channel regression (see Self-implemented Signal Processing: “Multi-channel Regression Type A: Global Regression” section). Data were acquired with the NIRStar Software version 14.0 (NIRx Medical Technologies LLC, Berlin, Germany) at a sampling rate of 7.81 Hz.

We calculated a spatial sensitivity profile based on the Monte Carlo photon migration forward modeling with 10^7^ photons using the AtlasViewer software (part of HOMER2 software package^1^, Huppert et al., 2009; Aasted et al., 2015) to assure that the chosen probe placement is selective for the measurement of the according ROIs (see Figure 1B). This proved that the selected probe setup allowed for measuring fNIRS signals that at least partly originate from changes in the cerebral compartment of both ROIs.

### Experimental Protocol

Prior to the experiment, the head circumference, nasion-inion length and ear-to-ear distance (between preauricular points) of the participants were measured. Participants were then seated on a chair, the cap was positioned on their head according to guidelines of the International 10/20 system (Chatrian et al., 1985) and the optodes were inserted into the cap on the defined positions. After mounting their non-dominant, left hand into the Amadeo device, subjects were instructed to sit still during the data acquisition to avoid motion artifacts in the fNIRS signals.

**Figure 2:**
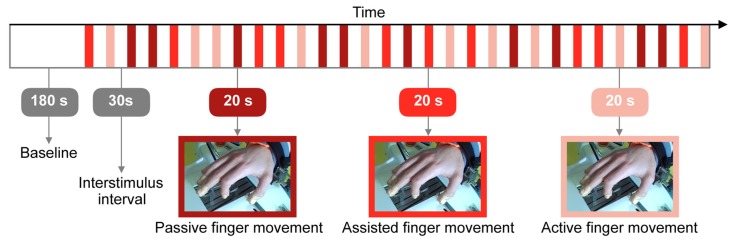
Visualization of the research protocol involving three different types of tasks: passive, assisted and active finger movement. The whole experiment lasted 27.5 min.

The task started with a resting state baseline of 180 s. Figure 2 displays the protocol which comprised three conditions: (i) “Passive finger movement”: Passive flexion and extension of the index finger, movement only by the Amadeo device at a frequency of 1 Hz (unidirectional, i.e., 1 s bending and then 1 s extending); (ii) “Assisted finger movement”: Assisted index finger movement: the Amadeo device guiding/initiating the movement, subjects instructed to perform the movement along with Amadeo at a frequency of 1 Hz; and (iii) “Active finger movement”: Self-paced index finger movement (at ~1 Hz) while mounted within the Amadeo device with no guidance (there was a slight resistance due to the static friction of the rails).

We used a randomized block design (Urbaniak and Plous, 2013) with 10 repetitions of each condition. Throughout the experiment, all other fingers remained at a constant position and 5 s before the beginning of each trial, subjects were informed which condition they had to perform next.

The chosen paradigm was hypothesized to elicit at the most marginal cortical effects accompanied by gradually increasing systemic hemodynamics (due to the increased muscular contribution from passive to active; Leff et al., 2011).

### Signal Processing

Each of the four different signal processing methods had its own processing pipeline. Signal processing and data analysis were done with Matlab (Version R2013b, MathWorks, Natick, MA, USA) and custom-made scripts, as well as with a commercially available Matlab-based fNIRS freeware (nirsLAB, version v2016.05, NIRx Medical Technologies, Glen Head, NY, USA). Matlab codes are available from the corresponding author upon reasonable request.

**Figure 3:**
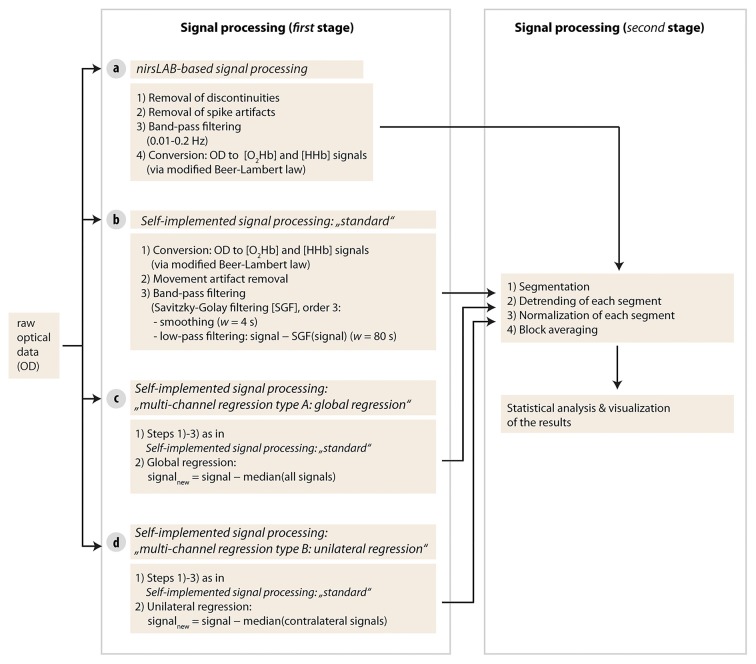
Visualization of the signal processing approaches used in the current study. Four different types of methods were used in stage one.

As a first step, raw optical density (OD) data were first imported into the commercial nirsLAB analysis software together with the probe information and all channels with a coefficient of variation of >15% (Piper et al., 2014) were excluded from further analysis (two channels in one subject and one channel in two other subjects). Then, data processing was done separately for each signal processing method according to the following pipelines (see visualization in Figure 3).

#### nirsLAB-based Signal Processing

1. The “remove discontinuities” option was selected. Thereby, the following steps are performed (adapted from the nirsLAB User Manual (Xu et al., 2016), *Chapter* 3.3 *Remove Discontinuities GUI*):
  (a) From the raw data (R) in each time series, the SD is computed.
  (b) For each pair of successive data values (R_i_ and R_i+1_), J_i_ = (R_i+1_ − R_i_)/SD is calculated.
  (c) All locations in the time series where J_i_ is ≥5 are identified.
  (d) Starting with R_i+1_, a constant value is subtracted from all data values following the jump: R’_k_ = R_k_ − (R_i+1_ − R_i_), with k = i + 1, …, n (n = final sample).
2. Motion artifacts (MA) were removed according to the nirsLAB user manual (Xu et al., 2016; *Chapter* 3.4 *Remove Spike Artifacts GUI)*. All channels were independently inspected by two scientists (MDP, RL) and MA were only marked as such, if both scientists agreed. All MA that were within stimulation periods or just before stimulation periods were selected and replaced with “random signals” (a set of random numbers that are sampled from a Gaussian distribution, with a SD equal to the average of the 4-second time intervals preceding and following the MA, and with a mean equal to the data value that immediately precedes the MA. Please note, that nirsLAB currently is programmed to replace the according period in all channels, not just in the selected one. On average, 2.3 MA (SD = 4.3) per subject were detected.
3. Data were band-pass filtered (cutoff frequencies: 0.01 Hz and 0.2 Hz; Koenraadt et al., 2014).
4. By applying the modified Beer-Lambert Law (Cope et al., 1988), the OD data were converted into the relative concentration changes in [O_2_Hb] and [HHb]. Thereby, we calculated the age-dependent differential path length factor (DPF) for each subject according to a previously published equation (Scholkmann and Wolf, 2013).
5. The baseline was defined as the period from second 120 to second 180 (see Figure 2). For the molar extinction coefficients of hemoglobin, we applied the spectrum as published by Schmitt (1986).
6. Data were exported and imported into Matlab for data analysis.

#### Self-implemented Signal Processing: “Standard”

1. OD data were converted into the relative concentration changes in [O_2_Hb] and [HHb] in the commercial nirsLAB analysis software identical to step 4 in “nirsLAB-Based Signal Processing” section.
2. Data were exported and imported into Matlab for further analysis.
3. MA removal was done with the spline interpolation method (Scholkmann et al., 2010). This method corrects each MA in the signal per channel by subtracting the interpolated MA segment from the MA segment and subsequently shifting the corrected segment to match it with the baseline of the segment preceding the MA segment. All MA were again independently checked by two scientists. On average, 10.5 MA (SD = 9.0) per subject were detected. The parameter values of the MA algorithm were chosen to be optimal for each data set (optimal was defined so that the application of the algorithm removed the MA efficiently while minimizing to induce new artifacts to the data). This optimization procedure was independent from further data analysis.
4. Band-pass filtering was done with the following approach: High-frequency noise of the signal was removed by applying a third degree Savitzky-Golay filter (Savitzky and Golay, 1964) with a window length of 4 s (Schafer, 2011); low-frequency noise was removed by subtracting the low-frequency trend, determined by applying the Savitzky-Golay filter with a window length of 80 s, from the data (Vrana et al., 2016). The Savitzky-Golay filter performs a local polynomial regression (in our case with order 3) and has the advantage of preserving the high-frequency structure of the data in a data-adaptive manner (Schafer, 2011).

#### Self-implemented Signal Processing: “Multi-channel Regression Type A: Global Regression”

1. OD data were converted into the relative concentration changes in [O_2_Hb] and [HHb] in the commercial nirsLAB analysis software identical to step 4 in “nirsLAB-Based Signal Processing” section.
2. Data were exported and imported into Matlab for further analysis.
3. MA were removed identical to step 3 in ‘Self-implemented Signal Processing: “Standard”’ section.
4. Filtering was done identical to step 4 in ‘Self-implemented Signal Processing: “Standard”’ section.
5. To reduce the contamination of the fNIRS signal from changes not due to neurovascular coupling, a multi-channel regression was applied employing the approach presented by Saager and Berger (2005). The corrected [O_2_Hb] and [HHb] signals were determined by removing a contamination surrogate signal from each channel. The surrogate signal thereby consisted of the median of all channels (see Figure 1, henceforward called “global regression”).

#### Self-implemented Signal Processing: “Multi-channel Regression Type B: Unilateral Regression”

1. OD data were converted into the relative concentration changes in [O_2_Hb] and [HHb] in the commercial nirsLAB analysis software identical to step 4 in “nirsLAB-Based Signal Processing” section.
2. Data were exported and imported into Matlab for further analysis.
3. MA were removed identical to step 3 in ‘Self-implemented Signal Processing: “Standard”’ section.
4. Filtering was done identical to step 4 in ‘Self-implemented Signal Processing: “Standard”’ section.
5. To reduce the contamination of the fNIRS signal from changes not due to neurovascular coupling, a multi-channel regression was applied employing the approach presented by Saager and Berger (2005). The corrected [O_2_Hb] and [HHb] signals were determined by removing a contamination surrogate signal from each channel. The surrogate signal thereby consisted of the median of all channels on the contralateral hemisphere to the respective channel (e.g., median of channels 1–8 to correct channel 15, see Figure 1, henceforward called “unilateral regression”).

#### Further Signal Processing: Detrending, Normalization and Block Averaging

After the individual processing per method, the datasets were segmented into intervals with a length of 20 s (stimulus duration) plus 5 s prestimulus baseline. Following this procedure, the whole dataset was segmented into 10 intervals per condition. These segments were then detrended by applying a linear regression to remove the slow physiological drift during each segment period. Furthermore, the segments were normalized by subtracting the median value of the prestimulus baseline from the signal in each segment in order to remove the intraindividual variance of the starting values. Then, block averages were calculated.

### Statistical Data Analysis

Relative changes of [O_2_Hb] and [HHb] were obtained by taking the median value of the period from 5 s after stimulus initiation to 15 s after stimulus initiation of each channel’s block average due to non-normal distribution of data. To test for differences across signal processing methods, a Friedman test was used accordingly. Alpha was set to 0.05. We additionally calculated topographic maps of the hemodynamic states with the commercial nirsLAB analysis software (nirsLAB User manual, *Chapter 5.3 Map Hemodynamic States: All States*, Xu et al., 2016) to provide a visual comparison between different signal processing methods.

## Results

**Figure 4:**
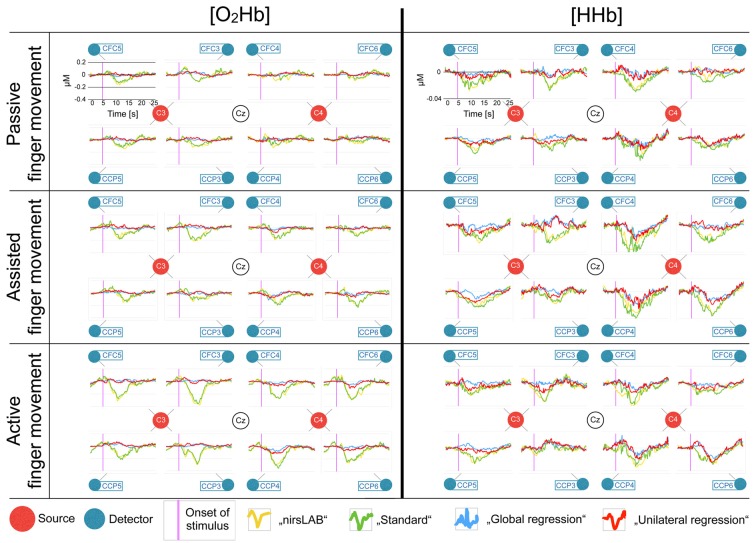
Block averages of each channel, separately for [O_2_Hb] and [HHb]. All eight recorded channels of the motor cortex are displayed in a top-view topographical organization around the left (C3) and the right motor cortex (C4). The labels in the red dots/next to the blue dots indicate 10/20 EEG positions. Shown are block averages of the data analyzed with nirsLAB-based signal processing (yellow curves), with the self-implemented signal processing: “standard” (green curves), with the self-implemented signal processing: “multi-channel regression type A: global regression” (blue curves), and with the self-implemented signal processing: “multi-channel regression type B: unilateral regression” (red curves). The x-axes display the time from 0 s to 25 s (purple line indicates stimulus initiation) and the y-axes display the relative hemoglobin concentration (0 corresponds to the median baseline), whereby the scales were kept constant for all curves in [O_2_Hb] and [HHb], respectively, to facilitate comparison (according to the axes in each top left chart).

Figure 4 shows the block averages of [O_2_Hb] and [HHb] and allows for a visual comparison of the outcome of the different signal processing methods. Generally, an increase in response amplitude could be seen when subjects increased their active participation (from passive via assisted to active finger movement). However, the direction of the response seems contrary to what is published in literature (when looking at the yellow and green traces in Figure 4; Perrey, 2008; Leff et al., 2011; Scholkmann et al., 2014). Furthermore, there is no clear lateralization of the response.

**Figure 5:**
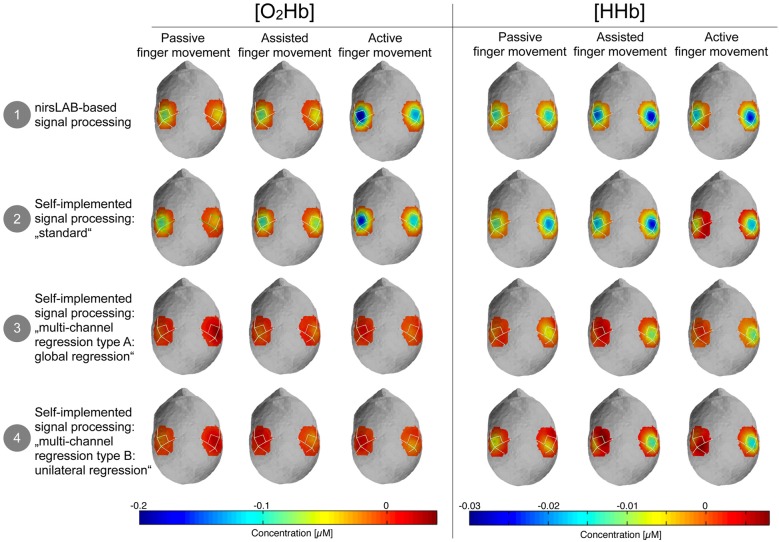
Topographic maps of the relative changes in amplitude of [O_2_Hb] and [HHb] compared to baseline. Blue areas represent channels where the amplitude was lower compared to baseline and dark red channels represent channels where the amplitude was larger compared to baseline. Please note that orange-red areas indicate no change compared to baseline.

Figure 5 shows the topographic maps of the relative changes of [O_2_Hb] and [HHb] in response to the stimulation, compared to baseline. It highlights that there is a visually discernible difference between signal processing methods that incorporate a multi-channel regression and those that do not.

**Figure 6:**
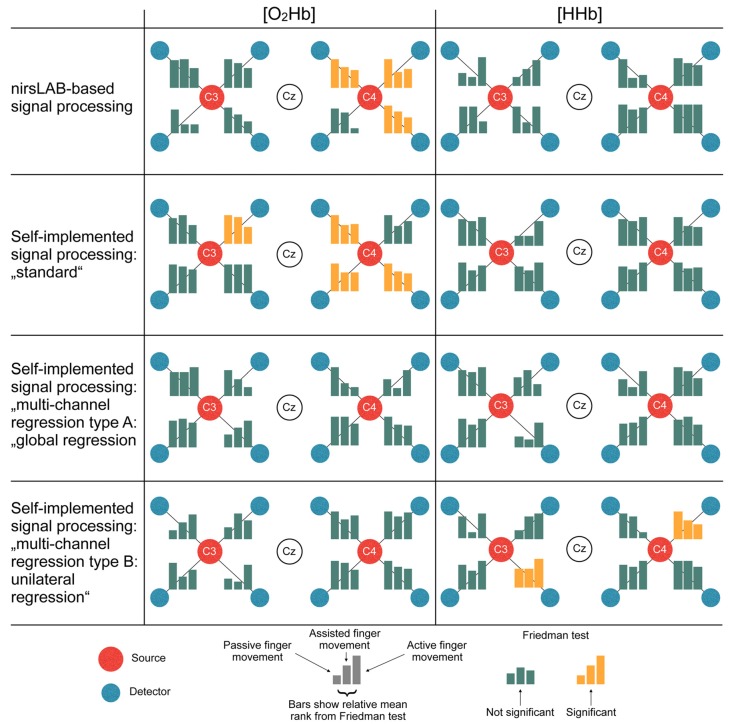
Statistical consequences of applying different signal processing methods. The bars represent the relative mean ranks from the non-parametric Friedman test (corresponds to the parametric repeated measures analysis of variance (ANOVA)) per channel in a top-view topographical organization around the left (C3) and the right motor cortex (C4). The labels in the red and white dots indicate 10/20 EEG positions. Yellow bars indicate that the Friedman test was significant, thus showing a statistical difference between the signal processing methods.

Figure 6 displays the statistical consequences of applying different signal processing methods. Each signal processing method leads to a different set of statistically significant channels. Thereby, especially the differences between methods with vs. methods without multi-channel regression become apparent. While the former led to 3–4 significant channels in [O_2_Hb] and 0 significant channels in [HHb], analyses with regression yielded 0 significant channels in [O_2_Hb] and 0–2 significant channels in [HHb].

## Discussion and Conclusions

Over the course of the last few years, a large body of evidence has been published regarding the contamination of the cortical hemodynamic signal acquired by fNIRS by cerebral and extracerebral hemodynamics not due to neurovascular coupling (Saager et al., 2011; Takahashi et al., 2011; Kirilina et al., 2012; Yamada et al., 2012; Gagnon et al., 2014; Tachtsidis and Scholkmann, 2016), and some were even specifically addressed to researchers new to the field (Orihuela-Espina et al., 2010; Leff et al., 2011). Nevertheless, especially in the fields of rehabilitation research, psychology and sport science, many articles were recently published that ignore the issue of signal contamination (suspected examples in the field of rehabilitation research: Basso Moro et al., 2014; Chang et al., 2014; Jang et al., 2015; Maidan et al., 2015, 2016; Al-Yahya et al., 2016; Mahoney et al., 2016; Nieuwhof et al., 2016; psychology: Bigliassi et al., 2014; Balconi et al., 2015; Lukanov et al., 2016; and sport science: Byun et al., 2014, 2016; Ono et al., 2015). Possible reasons include for example lacking knowledge, or technical limitations of the instrumentation (e.g., short-distance measurements are not possible due to fixed probe holders). In most of these articles, analyses were done using the software provided by the manufacturers of the used fNIRS devices, possibly applying the default configuration regarding filtering or artifact removal. To simulate the consequences of such an approach, we compared the standard processing method (nirsLAB) of the manufacturer of our fNIRS device without short-distance channels to three alternatives. We thereby used data from a sample of healthy individuals performing a motor task which we expected to lead only to a minor task-related cortical activation due to neurovascular coupling. However, it still elicited gradually increasing systemic hemodynamics due to the increasing muscular contribution. This can be seen in Figure 4 (yellow and green traces), where, especially in [O_2_Hb], an increase in response amplitude can be seen from passive to active finger movement. This increase, however, is only visible, when no multi-channel regression (blue trace) was applied. Obviously, the choice of the signal processing method had a considerable impact on the statistical results and consequently also on the conclusions one can draw from these results.

### Statistical Consequences

#### “nirsLAB” vs. “Self-implemented Signal Processing”

The publicly and freely available commercial nirsLAB analysis software is a rather comprehensive fNIRS analysis package. However, nirsLAB has only limited filtering and artifact removal options, as becomes apparent from the manual: “The components of Data Preprocessing (Chapter 3) allow you to: delete experimentally irrelevant time intervals from data; remove artifacts (“steps” and “spikes”) from data; and apply band-, low- or high-pass frequency filters to exclude experimentally irrelevant frequency bands” (from the nirsLAB User Manual, *Chapter 1.4.4.2 Data Preprocessing*, Xu et al., 2016). To investigate whether these limitations had an influence on the results, we compared nirsLAB to a self-implemented data-adaptive filtering and artifact removal method. While the visual comparison did not show distinct differences (Figures 4, 5), the statistical comparison presented a discrepancy between the two approaches. This finding stresses the importance of data filtering and artifact removal, as already repeatedly reported in literature (Zhang et al., 2009; Scholkmann et al., 2010, 2014; Brigadoi et al., 2014; Tak and Ye, 2014), and highlights the need for signal processing standards in the field.

#### Methods with vs. Methods without Multi-channel Regression

As already noted above, there were distinct differences between methods incorporating multi-channel regression and those without multi-channel regression (Figure 6). We speculate that the multi-channel regression reduced the sensitivity of fNIRS to measure changes in the hemodynamics not due to neurovascular coupling (Saager and Berger, 2005; Zeff et al., 2007), but this would have to be verified in a study using actual short-distance channels. Accordingly, when using a commercial device that by default does not offer the possibility of multi-distance measurements, it is strongly recommended to modify the device (as done e.g., in the article of Vrana et al., 2016) or to take the lack of multi-distance measurements into account by adjusting the signal processing appropriately as suggested in literature (Zhang et al., 2005; Kohno et al., 2007; Santosa et al., 2013; Tak and Ye, 2014).

#### Unilateral vs. Global Multi-channel Regression

We selected two different signals as a contamination surrogate: (i) the median of all channels (“global regression”); and (ii) the median of all channels on the opposite hemisphere (“unilateral regression”). The idea behind this approach is to eliminate the task-related physiological noise from the signal that is suspected to be similar in (half of) all channels (assuming that this component is mainly induced by systemic hemodynamics; Saager and Berger, 2005). This should increase the sensitivity of fNIRS to changes in the cortical compartment (Saager and Berger, 2005; Zeff et al., 2007).

The unilateral regression is supposed to show its strengths when being applied to the active hemisphere (contralateral to the moving finger), as the opposite hemisphere then presumably only shows minimal task-related cortical activation, thus reducing the probability of removing components of functional brain activity. On the other hand, when being applied to the non-active hemisphere (ipsilateral to moving finger), the surrogate signal might contain large components of functional brain activity which would then be eliminated from the respective channels.

The global regression might capture all the hemodynamic changes not due to neurovascular coupling happening in both the extracerebral (scalp blood flow) as well as the cerebral compartment (e.g., changes due to blood pressure or respiration (CO_2_ concentration in the blood)). The global regression is a rather blunt approach and, together with the unilateral regression, might be used only if no multi-distance measurements are possible. However, semi-simulations should first verify the usefulness of these approaches in improving the data quality of clinical measurements.

#### Shape of fNIRS Block Averages

We expected that the tasks would lead at the most to marginally detectable cortical activation, as the movements were very slow and minimal, and required no mental effort (as opposed to sequential finger tapping for instance; Leff et al., 2011). The results in Figures 4, 5 (“global regression”) seem to support this hypothesis, as neither [O_2_Hb] nor [HHb] show a distinct activation pattern as known from literature (Perrey, 2008; Leff et al., 2011; Scholkmann et al., 2014). Even though the actual response to the task is secondary, since we were mainly interested in the comparison between signal processing methods, the results of the analyses without regression nevertheless were unanticipated. We hypothesized an actual increase in [O_2_Hb] and a decrease in [HHb] in these signal processing methods, since we expected an increase in scalp skin blood flow to happen. However, a hemodynamic task-evoked response due to neurovascular coupling seems absent. Further studies are needed to clarify this phenomenon.

## Limitations

First of all, our measurement setup did not include multi-distance measurement. Therefore, we can only estimate which channels were effectively active. However, this article was written to convince researchers new to the field of fNIRS to implement mechanisms to avoid a contamination of the signal to an extent which might lead to an invalid interpretation of the results. Therefore, we decided to use a setup similar to that presumably used by such researchers. Furthermore, the calculation of the global regressor includes the signal of target channels where an actual task-evoked hemodynamic response due to neurovascular coupling is expected. In case of a strong response, which was not the case in our study, this could negatively influence the efficacy of the regression. In that case, a leave-one-out method would be more appropriate.

It is important to say that we applied the regression analyses exploratively. The chosen approach might not be generalizable to other measurement setups and it needs to be verified first in a setup containing short-channels. However, the focus of the project was to highlight possible consequences of applying different methods.

Even though we tried to objectify the process of selecting motion artifacts (e.g., by choosing a consensus approach), it is still possible that this process led to the exclusion of physiological signals or contributed to the shown differences between methods (see “nirsLAB” vs. “Self-implemented Signal Processing”section). Additionally, including random signals or applying the spline interpolation method add non-physiological data which are also actually artifacts. Furthermore, the motion artifact detection method applied in the self-implemented signal processing (Scholkmann et al., 2010) is more sensitive and allows for a more fine-grained detection of motion artifacts compared to the manual method applied within nirsLAB. This fact also contributes to the differences in statistical outcome.

Furthermore, we applied a block design with a constant duration of task and rest intervals (Figure 2). This is known to cause artifacts as well that are not eliminated by the filtering procedures we used (Scholkmann et al., 2014). Further studies are necessary to determine to which extent these artifacts can be avoided by using block designs with variable block lengths.

To help maintaining the focus of the participants, they were given information on the type of condition shortly before its start. This might have led to an anticipation effect which we have not controlled for. Nevertheless, it is unlikely that this influenced the results on statistical differences between different signal processing methods.

Finally, we decided to analyze the data with simple block averages. General linear models offer additional possibilities to reduce the signal contamination (e.g., by adding simultaneously recorded physiological signals, like heart rate, blood pressure, or breathing parameters, as independent factors to the model). However, we kept it deliberately simple to specifically address the target audience of researchers new to the field of fNIRS.

## Conclusion

Even though the field of fNIRS just turned 20 years old (Boas et al., 2014), a consensus on a common signal processing and data analysis pipeline is still lacking. On the one hand, established and experienced research groups regularly publish new recommendations which eventually should build the basis for such a consensus. On the other hand, many new researchers enter the field, creating a significant knowledge gap which is hard to bypass. This study highlights the importance of correcting for the contamination of the measured fNIRS signals by task-evoked (or stimulus-evoked) hemodynamic changes not due to neurovascular coupling. Failing to do so can have a significant influence on statistical results and possibly on their interpretation. Accordingly, we recommend to use standard signal processing methods as provided by the manufacturers only when having an advanced understanding of every performed step. Furthermore, when lacking the possibility of applying multi-distance measurements, we recommend clinical users to adopt according signal processing methods, as proposed in literature.

## Data Availability

The Matlab codes used are available from the corresponding authors upon reasonable request.

## Author Contributions

MDP and RL conceived, planned the experiments and carried out the data preprocessing. MDP carried out the experiments. FS and RL planned, carried out the data analysis and contributed to the interpretation of the results. RL took the lead in writing the manuscript. All authors provided critical feedback and helped shape the research, analysis and manuscript.

## Conflict of Interest Statement

The authors declare that the research was conducted in the absence of any commercial or financial relationships that could be construed as a potential conflict of interest.

## Footnotes

^1^http://www.nitrc.org/projects/homer2
